# Spectrum of Histomorphologic Findings in Liver in Patients with SLE: A Review

**DOI:** 10.1155/2014/562979

**Published:** 2014-07-21

**Authors:** Shrruti Grover, Archana Rastogi, Jyotsna Singh, Apurba Rajbongshi, Chhagan Bihari

**Affiliations:** Department of Pathology, Institute of Liver and Biliary Sciences D-1, Vasant Kunj, New Delhi 110070, India

## Abstract

Collagen vascular diseases (CVDs) like systemic lupus erythematosus (SLE), rheumatoid arthritis, Sjogren syndrome (SS), and scleroderma are immunologically mediated disorders that typically have multisystem involvement. Although clinically significant liver involvement is rare, liver enzyme abnormalities are common in these patients. The reported prevalence of hepatic involvement in SLE, histopathologic findings, and its significance is very variable in the existing literature. It is important to be familiar with the causes of hepatic involvement in SLE along with histomorphological features which aid in distinguishing hepatitis of SLE from other hepatic causes as they would alter the patient management and disease course. Histopathology of liver in SLE shows a wide morphological spectrum commonly due to a coexisting pathology. Drug induced hepatitis, viral etiology, and autoimmune overlap should be excluded before attributing the changes to SLE itself. Common histopathologic findings in SLE include fatty liver, portal inflammation, and vascular changes like hemangioma, congestion, nodular regenerative hyperplasia, arteritis, and abnormal vessels in portal tracts.

## 1. Introduction

Systemic lupus erythematosus (SLE) is a chronic autoimmune disease with features of multisystem involvement and diverse clinical and serological manifestations, mostly affecting women during the child bearing age. Hepatic involvement is usually subclinical and has been demonstrated by many studies [1]. Clinically significant hepatic dysfunction is generally considered uncommon in SLE and treatment with hepatotoxic drugs or viral hepatitis has usually been implicated as the most pertinent causes for such unusual complications [2]. Commonly recognised features include mild elevation of liver enzymes and nonspecific histological features. Liver involvement may vary from a mild asymptomatic elevation of liver transaminases to a fulminant hepatitis rarely. Liver involvement as a manifestation of underlying SLE requires cautious exclusion of hepatotoxic drugs or coincident viral hepatitis.

Hepatic involvement in SLE could be due to a wide range of factors such as drug induced damage, steatosis, viral hepatitis, vascular thrombosis, and overlaps with autoimmune hepatitis (AIH) or due to SLE itself. It is important to differentiate between the above causes of hepatic involvement as they alter the disease course and management [3]. A range of studies evaluating the causes of liver involvement in SLE have been conducted and results of histopathological features have been inconsistent. A review of histomorphological features of liver in patients with SLE is presented.

## 2. Methodology

We searched the literature on PubMed database with the following keywords: “SLE, liver histopathology” which showed a total of 168 studies. Out of these, all studies which evaluated the causes of liver dysfunction in SLE patients by histopathology were shortlisted. These amounted to a total of 36 studies. All studies where histopathology of liver was not available were excluded from the histological spectrum in the present review.

All the 36 studies were assessed on methodology. These included patients diagnosed with SLE according to revised criteria proposed by American College of Rheumatology. The criteria for ascertaining lupus as the primary cause of liver dysfunction were based on exclusion of all other possible causes of liver dysfunction such as viral hepatitis, drug induced liver damage, nonalcoholic steatohepatitis, and any known underlying vascular or haematological disorder. Viral hepatitis excluded by negative serology and drug induced toxicity was evaluated by history and histopathological features in these studies. Patients of SLE who were on drugs/steroids as a part of the treatment of underlying autoimmune disease were included, excluding patients on any other kind of drug treatment. Patients who had known association with vascular thrombosis or any haematological disorder were excluded in the present review. Patients with features of metabolic syndrome were also excluded. A radiological study reporting benign vascular lesions in SLE was also included although no histological confirmation was available in this study.

**Figure 1:**
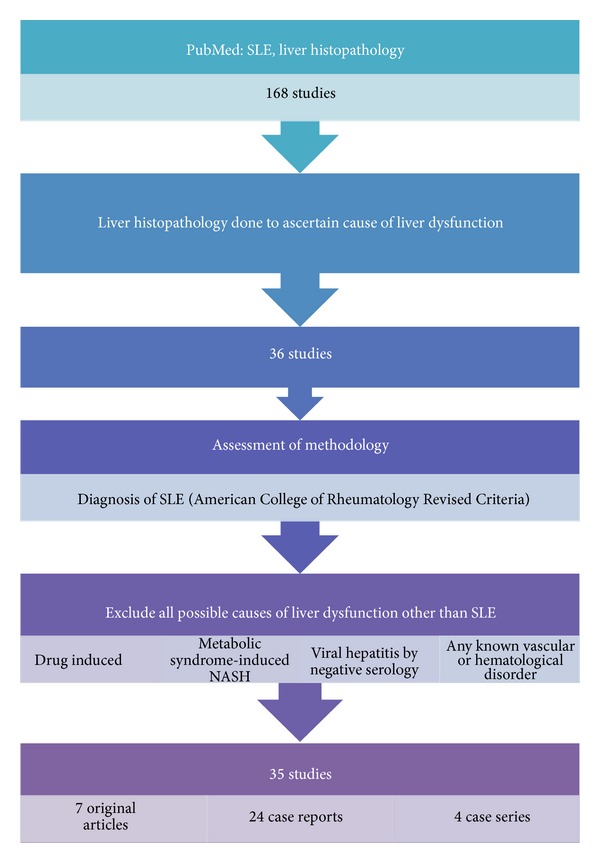
Summarized methodology.

After applying the above standard the total number of studies amounted to 35 comprising 7 original articles, 24 case reports, and 4 case series. Combining all the above studies histopathological spectrum of liver in SLE was studied in 293 patients. Few cross-references in these articles were also studied and included in the review (methodology summarized in Figure 1).

## 3. Review

### 3.1. Subclinical Liver Disease

Subclinical liver disease is a common phenomenon in SLE mostly manifesting as abnormal liver function test. The reported values of liver enzyme elevation range up to 55% [4]. Recently a study by Vaiphei et al. reported a much higher value of 81% of SLE patients showing raised transaminases [5]. A prospective study reported liver enzyme elevations in 23% out of which in 8% cases the elevations were unexplained. Liver tissue available from 14 patients revealed no significant lesions. This study suggested that subclinical liver disease is a manifestation of SLE [1].

The reported frequency of hepatic involvement in SLE ranges from 8 to 23% [4]. Clinical examination may show a palpably enlarged liver in 33% cases [5].

### 3.2. Biochemical Abnormalities

Biochemical abnormalities are typically mild and show transient elevation of liver enzymes. The histologic abnormalities in most patients are commonly nonprogressive. Such biochemical and histologic findings can be attributed to the underlying autoimmune condition and require no specific management. Patients with a coexisting primary liver disease show a more persistently deranged LFT and further workup using serologic tests, imaging studies, and liver biopsy is needed to precisely identify the cause of liver test abnormalities [6].

### 3.3. Histopathology of Liver in SLE

Pathologically, a wide variety of lesions have been described in the hepatic parenchyma of patients diagnosed with SLE. Liver histology can show steatosis, portal and lobular inflammation, hepatic granulomas, centrilobular necrosis, microabscesses, haemochromatosis, cholestasis, nonspecific reactive changes, and infrequent cirrhosis [4].

Histopathological findings are summarized in Table 1.

#### 3.3.1. Portal Changes

**Figure 2:**
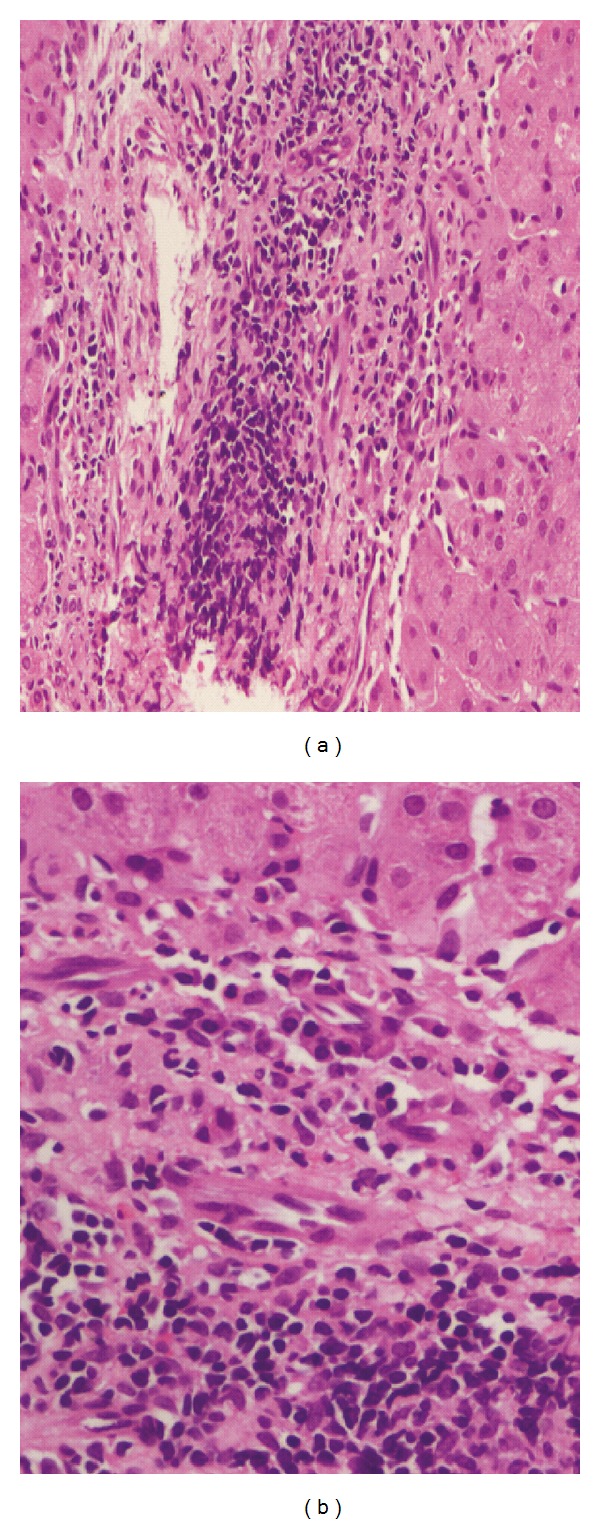
(a) Portal inflammation with lymphoid aggregate (H&E stain; 200x). (b) Portal inflammation comprising of numerous plasma cells with interface activity (H&E; 400x).

The various alterations seen in portal areas include portal inflammation, abnormal vessels, interface hepatitis, chronic persistent hepatitis, nonspecific reactive hepatitis, portal tract fibrosis, and periductal fibrosis. Portal tract inflammation was a relatively common histopathological finding [4, 5, 7–9]. Vaiphei et al. studied 21 autopsy cases of SLE and portal tract inflammation was seen in 14 patients. The inflammation was mild to moderate and comprised of mainly lymphocytes, plasma cells, neutrophils, and occasional eosinophils (Figure 2). No lymphoid follicle, bile duct epithelial cell injury, plasma cell dominant infiltration, or hepatocyte rosette formation was seen in these cases [5]. Along with this interface hepatitis has been also reported by some authors [4, 5, 7].

#### 3.3.2. Lobular Changes

**Figure 3:**
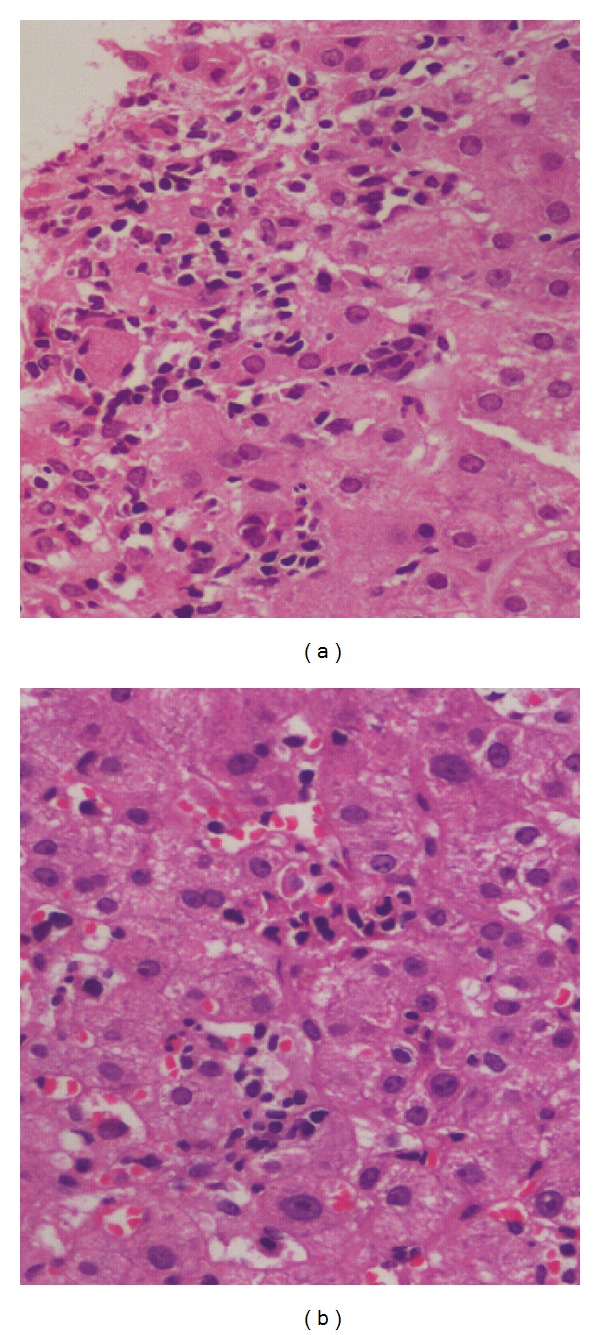
(a) Foci of confluent necrosis infiltrated by inflammatory cells (H&E; 400x). (b) Microabscesses in hepatic lobule (H&E stain; 400x).

**Figure 4:**
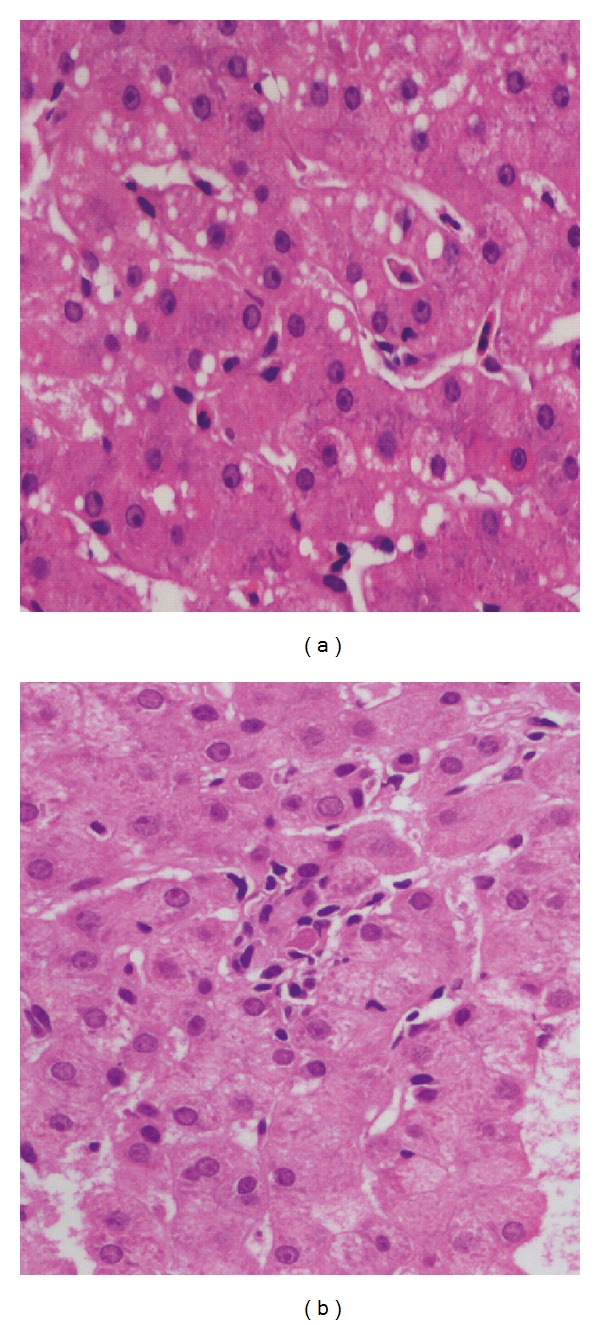
(a) Mild macrovesicular steatosis in hepatocytes (H&E stain; 400x). (b) Small focus of lobular inflammation (H&E; 400x).

Lobular changes were not as frequent as portal changes. These included lobular inflammation, steatosis, and focal necrosis [5, 8] (Figure 3). Hydropic degeneration of hepatocytes has also been described by one author in 8 of the 47 SLE cases studied [8]. Hepatocytic steatosis was a fairly common morphologic observation in many studies and it could not be conclusively attributed to lupus alone since similar changes can occur due to steroid therapy which is commonly prescribed to these patients [4, 7–10] (Figure 4). Matsumoto et al. studied 52 livers from patients with systemic lupus erythematosus and found fatty liver in a significant number of patients (38/52). They considered steatosis as a finding specific to SLE. Exposure to steroids in these patients was a significant etiologic factor in development of fatty liver [10]. There have been rare case reports of secondary amyloidosis of liver in patients of long-standing SLE [11].

#### 3.3.3. Vascular Changes

A wide morphologic spectrum of vascular changes have been described by many authors which include hepatic congestion, abnormal vessels in portal tracts, hemangioma, peliosis hepatis, arteritis, and occasionally infarct due to arteritis. Rarely hepatic artery aneurysms [12] and spontaneous hepatic rupture due to arteritis of hepatic arteries have been also been reported [13]. Matsumoto et al. found hepatic congestion to be the commonest (76%) in 52 livers studied, along with arteritis (22%), peliosis hepatis (11.5%), hemangioma, and nodular regenerative hyperplasia in three patients each (5%) [10]. Fatty change was a significant finding noted in 73% of cases. Their data suggested that arteritis of liver in SLE was more common than that reported previously and one patient developed hepatic infarction as a complication induced by arteritis. Congestion was linked to acute terminal illness.

Vaiphei et al. studied 21 SLE patients with no known association with chronic liver disease or vascular thrombosis or hematological disorder and found diffuse nodular regenerative hyperplasia of liver (NRHL) in a significant proportion of cases (43%) with some portal tracts showing mild-to-moderate chronic inflammation with occasional bridging fibrosis [5]. This finding of NRH was more frequent in their study than previous reported series [14, 15]. Matsumoto et al. reported numerous abnormal thin-walled vessels in intermediate- and small-sized portal tracts with no vascular occlusion or inflammation. These vascular channels were seen involving about 40% of intermediate- and small-sized portal tracts entrapping bile ducts and hepatic arteries with a variable amount of collagen in between. Etiopathogenesis for NRHL in SLE is an immune complex deposit in small vessels resulting in obliterative venopathy. Obliterative fibroinflammation of the terminal portal tract was observed in all NRHL cases in this study. The proposed pathogenesis of NRHL is a small vessel vasculitis producing hepatocytic atrophy associated with compensatory hyperplasia [16].

The prevalence of liver hemangioma was 54.2% in 1 study involving 35 patients of SLE compared to 14% in general population. The above authors also reported a case of Budd-Chiari syndrome with NRH and NRH associated with hepatic hemangioma both in patients hospitalized for abdominal symptoms, suggesting that vascular liver diseases should be specifically investigated in this population, but this study lacked the histological confirmation of hemangioma in most patients as imaging modalities were used for diagnosis [17].

#### 3.3.4. Biliary Changes

Biliary changes are not frequent in SLE. Histopathology has revealed cholestasis, cholangiolitis, and periductal fibrosis in few cases and acute cholestatic hepatitis has been reported rarely [5, 10]. Runyon et al. demonstrated a peculiar form of cholestasis resembling a canalicular cast of bile in three of four SLE patients with cirrhosis [7]. The canalicular cast of bile is a form of cholestatic liver cell rosettes and can be found in any long-standing canalicular cholestasis. Occasional case report describes hemobilia in liver biopsy in a patient who developed acalculous cholecystitis [18].

#### 3.3.5. Advanced Fibrosis/Cirrhosis

Portal tract fibrosis and bridging fibrosis were a common observation reported by many [5, 7, 9] with cirrhosis in few and progression to liver failure was reported by Runyon et al. in 3 cases [7]. This was the first study to highlight that severe and fatal liver disease can occur in SLE due to liver failure. Vaiphei et al. observed portal tract fibrosis in 76% and bridging fibrosis in 42% of the cases with progression to cirrhosis in minority of cases by various authors [3, 7, 8, and 9]. Matsumoto's autopsy registry review of 1468 patients suggested an incidence of chronic hepatitis in 2.4% of SLE patients with 1.1% progressing to cirrhosis [10].

#### 3.3.6. AIH and SLE Overlap

Differentiating between liver involvement in CVDs and overlap syndrome with AIH could be difficult given their common clinical and serologic manifestations. Differentiating features for AIH from SLE-related liver disease are portal and periportal inflammation, piecemeal necrosis with dense lymphoid infiltrates, dominant portal tract plasma cell infiltration, hepatocyte pseudorosette formation whereas lupus-associated hepatitis shows predominantly lobular involvement with mild lobular inflammation and no piecemeal necrosis [19].

One study suggested use of antiribosomal P antibodies to distinguish between the two entities as they are not found in patients with AIH but are present in a significant proportion of patients with lupus-associated hepatitis [20].

Distinguishing the two entities is important as the diagnosis has important prognostic and therapeutic implications, where lupus-associated hepatitis has a more benign course and does not require corticosteroid therapy.

### 3.4. Coexistent Liver Pathologies in SLE

Liver disease in SLE could be the result of a variety of factors including fat infiltration, drug toxicity, coexisting viral hepatitis, vascular thrombosis, and overlap with autoimmune hepatitis (AIH) or of SLE itself. It is important to exclude these coexistent pathologies affecting the liver which may occur concurrently or sequentially, as they alter the disease course and management (summarized in Table 2).

Excessive fatty infiltration is a common finding in tissue and may be attributed either to the disease process itself or to the steroid treatment. Drug induced hepatitis is a relatively common cause of liver dysfunction in this group of patients which should therefore be excluded first before ascribing the cause of liver dysfunction as SLE. According to Gibson and Myers, a significant number of patients (14 out of 45) of SLE with elevated liver enzymes had drug induced hepatitis [4]. After excluding other nonhepatic causes 19 patients had enzyme elevation due to SLE itself. Van Hoek studied the spectrum of liver disease in SLE patients and suggested drug induced hepatitis as the most common cause of liver dysfunction [21].

A wide spectrum of primary liver diseases in systemic lupus erythematosus have been demonstrated by various authors and include viral hepatitis, autoimmune hepatitis, primary biliary cirrhosis, granulomatous hepatitis, giant cell hepatitis, chronic hepatitis with IgA or IgD deficiency, porphyria or idiopathic portal hypertension, and rarely lymphoma. Few infections like* Cryptococcus*,* Candida*, and* Listeria monocytogenes* have also been described [3, 7, 20, 22–24].

Chowdhary et al. retrospectively reviewed 40 cases of SLE to determine the presence of end-stage liver disease in patients with SLE and found that all except 6 cases had multiple causes of liver involvement other than SLE. These included drug induced (*n* = 4), viral hepatitis (hepatitis B or C and cytomegalovirus; *n* = 8), nonalcoholic fatty liver disease (NAFLD; *n* = 8), autoimmune hepatitis (AIH; *n* = 6), primary biliary cirrhosis (PBC; *n* = 3), and liver involvement from infection (2), cryptogenic cirrhosis (2), lymphoma (1), and indeterminate (6). Eight patients died. Mortality was not directly related to liver disease in any patient. They concluded that complications of portal hypertension, cirrhosis, and hepatic encephalopathy are rare manifestations of SLE unless coexistent liver disease such as NAFLD, viral hepatitis, or AIH is present [3].

There is no definite pathogenetic mechanism to explain the above histopathologic findings in SLE patients. Hepatic hemangioma is a benign vascular tumor and reported as a common finding in SLE. Hemangiomas are formed as a result of imbalance between proangiogenic and antiangiogenic factors [25]. Estrogen overactivity promotes angiogenesis and is exemplified by increased incidence of hemangiomas during pregnancy and estrogen therapy. Berzigotti et al. hypothesised that since SLE patients have increased circulating estrogen levels and angiogenic factors like vascular endothelial growth factor (VEGF), this proangiogenic state might lead to development of liver hemangiomas in SLE [17]. Nodular regenerative hyperplasia is proposed to occur due to a small vessel vasculitis leading to atrophy with compensatory hyperplasia [5].

## 4. Conclusion

To summarise, histopathology of liver in SLE shows a wide morphological spectrum commonly due to a coexisting pathology. Drug induced hepatitis, viral etiology, and autoimmune overlap should be excluded before attributing the changes to SLE itself. Common histopathologic findings in SLE include fatty liver, portal inflammation, and vascular changes like hemangioma, congestion, nodular regenerative hyperplasia, arteritis, and abnormal vessels in portal tracts. Progression to cirrhosis is not very common, but liver failure can occur rarely.

## Figures and Tables

**Table 1:** Spectrum of salient histomorphological findings in liver biopsy in patients of SLE.

	Portal inflammation
Portal changes [4, 5, 7–10]	Interface hepatitis
	Chronic persistent hepatitis
	Portal tract fibrosis
	Cholestasis
	Periductal fibrosis
	Cholangiolitis

Lobular changes [5, 8]	Lobular inflammation
	Focal necrosis
	Steatosis
	NAFL
	Hydropic degeneration

Fibrosis [3, 5, 7–9]	Bridging fibrosis
	Cirrhosis

Vascular changes [5, 10, 12–15, 17]	Abnormal vessels in portal tracts
	Arteritis
	Infarct due to arteritis
	Nodular regenerative hyperplasia
	Haemangioma
	Hepatic congestion
	Peliosis hepatis

**Table 2:** Associated liver pathology in patients of SLE.

	Drug induced hepatitis
Associated pathology [3, 7, 22–24]	Viral hepatitis
	Primary biliary cirrhosis
	Autoimmune hepatitis
	Lymphoma
	Granuloma
	Liver failure
	Infections
